#  Evaluation of the 2016–2020 regional tuberculosis response framework, WHO Western Pacific Region

**DOI:** 10.2471/BLT.20.268060

**Published:** 2021-03-02

**Authors:** Kerri Viney, Chris Lowbridge, Fukushi Morishita, Kalpeshsinh Rahevar, Kyung H Oh, Tauhid Islam, Ben J Marais

**Affiliations:** a Research School of Population Health, Australian National University, Canberra, Australian Capital Territory, 2600, Australia.; b Global and Tropical Health, Menzies School of Health Research, Charles Darwin University, Northern Territory, Australia.; c End TB and Leprosy Unit, World Health Organization Regional Office for the Western Pacific, Manila, Philippines.; d WHO Collaborating Centre for Tuberculosis and the Marie Bashir Institute for Infectious Diseases and Biosecurity, University of Sydney, Sydney, Australia.

## Abstract

**Objective:**

To assess the implementation of the *Regional framework for action on implementation of the End TB Strategy in the Western Pacific, 2016–2020* in countries and areas in the World Health Organization Western Pacific Region.

**Methods:**

We used a mixed methods approach to assess the framework’s measurable and perceived impact. We conducted an analysis of national tuberculosis strategic plans, a cross-sectional survey of senior staff of tuberculosis programmes, key informant interviews and some country case studies.

**Findings:**

Of the 37 countries and areas of the Western Pacific Region, 14 had a national tuberculosis strategic plan, including all countries and areas with a high incidence of tuberculosis. Most senior tuberculosis programme staff who responded to the survey (16/23) found the regional framework useful when developing their national targets and grant applications. Programmatic challenges identified included financing, human resources, public–private mix, active case finding, and paediatric and drug-resistant tuberculosis. Most of the 17 key informants thought that the regional framework’s categorization of actions (for all settings, for specific settings and for pre-elimination settings) was useful, but that the added value of the regional framework over other relevant documents was not obvious because of overlap in content.

**Conclusion:**

The regional framework influenced national level tuberculosis control planning and implementation in a positive way. A future regional framework should provide a longer-term strategic horizon and specifically address emerging trends and persistent problems faced by countries or areas of the region.

## Résumé

**Objectif:**

Évaluer la mise en œuvre du *Cadre d'action régional pour la mise en œuvre de la Stratégie visant à mettre un terme à la tuberculose dans le Pacifique occidental 2016–2020* dans les pays et territoires appartenant à la Région du Pacifique occidental de l'Organisation mondiale de la Santé.

**Méthodes:**

Nous avons adopté une approche de méthodes mixtes afin de calculer l'incidence mesurable et effective du cadre. Nous avons mené une analyse des plans stratégiques nationaux de lutte contre la tuberculose, une enquête transversale auprès des directeurs de programmes de lutte contre la tuberculose, des entretiens avec les principaux intervenants, et quelques études de cas concernant différents pays.

**Résultats:**

Sur les 37 pays et territoires de la Région du Pacifique occidental, 14 possédaient un plan stratégique national de lutte contre la tuberculose, y compris tous les pays où la maladie est encore très présente. La plupart des directeurs de programmes de lutte contre la tuberculose ayant répondu à l'enquête (16/23) ont jugé le cadre régional utile à l'élaboration de leurs objectifs nationaux et demandes de subventions. Plusieurs défis liés aux programmes ont été identifiés: financement, ressources humaines, mélange public-privé, dépistage actif des cas, mais aussi tuberculose pédiatrique et pharmacorésistante. La majorité des 17 intervenants clés interrogés estimaient que la classification des actions dans le cadre régional (pour toutes les phases, pour les phases spécifiques et pour les phases de pré-élimination) était judicieuse, mais que la valeur ajoutée du cadre régional vis-à-vis d'autres documents pertinents n'était pas toujours évidente en raison d'un recoupement des contenus.

**Conclusion:**

Le cadre régional a eu une influence positive sur la planification et la mise en œuvre de la lutte contre la tuberculose au niveau national. Le futur cadre régional doit fournir un horizon stratégique à plus long terme; il doit également tenir compte des nouvelles tendances et des problèmes persistants rencontrés par les pays et territoires de la région.

## Resumen

**Objetivo:**

Evaluar la aplicación del *Marco de acción regional para la implementación de la* e*strategia para detener la tuberculosis en el Pacífico Occidental*, 2016-2020 en los países y áreas de la Región del Pacífico Occidental de la Organización Mundial de la Salud.

**Métodos:**

Utilizamos un enfoque de métodos mixtos para evaluar el impacto medible y percibido del marco. Realizamos un análisis de los planes estratégicos nacionales contra la tuberculosis, una encuesta transversal del personal directivo de los programas contra la tuberculosis, entrevistas con informantes clave y algunos estudios de casos de países.

**Resultados:**

De los 37 países y áreas de la Región del Pacífico Occidental, 14 contaban con un plan estratégico nacional contra la tuberculosis, entre los que se encontraban todos los países con una alta incidencia de la enfermedad. La mayor parte del personal directivo de los programas de tuberculosis que respondió a la encuesta (16/23) consideró que el marco regional era útil a la hora de elaborar sus objetivos nacionales y sus solicitudes de subvención. Los retos programáticos identificados fueron la financiación, los recursos humanos, la combinación público-privada, la búsqueda activa de casos y la tuberculosis pediátrica y farmacorresistente. La mayoría de los 17 informantes clave consideraron que la categorización de las acciones del marco regional (para todos los entornos, para entornos específicos y para entornos de preeliminación) era útil, pero que el valor añadido del marco regional respecto a otros documentos pertinentes no era obvio debido al solapamiento del contenido**. **

**Conclusión:**

El marco regional influyó positivamente en la planificación y aplicación del control de la tuberculosis a nivel nacional. Un futuro marco regional debería proporcionar un horizonte estratégico a más largo plazo y abordar específicamente las tendencias emergentes y los problemas persistentes a los que se enfrentan los países y áreas de la región.

## ملخص

الغرض تقييم تنفيذ إطار العمل الإقليمي الخاص بتنفيذ استراتيجية القضاء على مرض السل في منطقة غرب المحيط الهادئ، من 2016 إلى 2020 في البلدان والمناطق في منطقة غرب المحيط الهادئ في منظمة الصحة العالمية.

الطريقة اتبعنا نهجًا يتضمن أساليب مختلطة لتقييم أثر إطار العمل القابل للقياس والملموس. أجرينا تحليلاً للخطط الاستراتيجية الوطنية لمرض السل، واستبيانًا للآراء يتضمن قطاعات متعددة من كبار موظفي أطقم العمل في البرامج الخاصة بالمرض، ومقابلات شخصية مع المبلغين الرئيسيين ودراسات حالة لبعض الدول.

النتائج من بين 37 بلد ومنطقة في منطقة غرب المحيط الهادئ، كان لدى 14 دولة خطة استراتيجية وطنية لمرض السل، بما في ذلك كل الدول ذات المعدل العالي للإصابة بالمرض. يرى معظم كبار موظفي أطقم العمل في البرامج الخاصة بمرض السل الذين شاركوا في استبيان الآراء (16/23) أن الإطار الإقليمي مفيد عند وضع أهدافهم الوطنية وطلباتهم للحصول على منح. كانت التحديات المحددة في البرامج تشمل التمويل، والموارد البشرية، والجمع بين القطاعين العام والخاص، والاكتشاف النشط للحالات، ومرض السل لدى الأطفال والمقاوم للعقاقير. كان معظم المبلغين الرئيسيين البالغ عددهم 17 يعتقدون أن تصنيف الإجراءات ضمن الإطار الإقليمي (بالنسبة لكل الأماكن وبالنسبة لأماكن محددة وبالنسبة لأماكن العزل المسبق) كان مفيدًا، لكن القيمة المضافة للإطار الإقليمي بالمقارنة مع المستندات الأخرى ذات الصلة لم تكن واضحة بسبب التداخل في المحتوى.

الاستنتاج حقق الإطار الإقليمي تأثيرًا إيجابيًا على التخطيط والتنفيذ لمكافحة مرض السل على المستوى الوطني. ينبغي أن يقدم الإطار الإقليمي المستقبلي أفقًا استراتيجيًا طويل الأمد ويتناول بشكل محدد التوجهات الناشئة والمشاكل المستمرة التي تواجهها بلدان أو أنحاء المنطقة.

## 摘要

**目的:**

旨在评估世界卫生组织西太平洋地区国家和区域*实施《西太区终止结核病策略 2016-2020 行动框架》*的情况。

**方法:**

我们采用混合方法来评估框架的可衡量和可感知的影响。我们对国家结核病战略计划进行了分析,对结核病项目高级工作人员进行了横断调查,对主要知情者进行了访谈以及针对一些国家案例开展了研究。

**结果:**

在 37 个西太平洋地区国家和区域中,14 个国家制定了国家结核病战略计划,包括所有结核病高发国家。参与调查的大多数结核病项目高级工作人员 (16/23) 认为,区域框架在制定其国家目标和经费申请时很有用。确定的规划挑战包括融资、人力资源、公私混合、主动病例发现以及小儿和耐药结核病。在 17 名主要知情者中,大多数人认为,区域框架对(针对所有背景、特定背景和消除前背景的)行动的分类很有助益,但由于内容重叠,区域框架相对于其他相关文件的附加价值并不明显。

**结论:**

该区域框架对国家结核病控制规划和实施产生了积极影响。未来的区域框架应提供较长期的战略视野,并具体处理该地区各个国家和区域面临的新趋势和长期问题。

## Резюме

**Цель:**

Оценить реализацию *регионального механизма действий по осуществлению стратегии по ликвидации туберкулеза в западной части Тихого океана в 2016 – 2020 гг.* в странах и районах, входящих в регион западной части Тихого океана в составе Всемирной организации здравоохранения.

**Методы:**

Авторы использовали смешанный методологический подход для оценки измеримого и наблюдаемого воздействия указанного механизма. Был выполнен анализ национальных стратегических планов по борьбе с туберкулезом, опрошены руководители противотуберкулезных программ в порядке одномоментного углубленного исследования, проведены интервью с основными информаторами, а также проведен разбор конкретных ситуаций на уровне стран.

**Результаты:**

Из 37 стран и районов западной части Тихого океана 14 имели национальные стратегические планы по борьбе с туберкулезом, в том числе все страны с высоким уровнем заболеваемости туберкулезом. Большинство руководителей противотуберкулезных программ, которые ответили на анкетирование (16 из 23), отметили, что региональный механизм был полезен для разработки их национальных целей и подачи заявок на гранты. Проблемы программного характера касались финансирования, кадров, смешанного общественного и частного характера услуг, поиска активных случаев заболевания, а также ситуации с туберкулезом у детей и с лекарственно-устойчивыми формами туберкулеза. Большинство из 17 основных информаторов полагало, что категоризация действий в рамках регионального механизма (для всех условий работы, для конкретных ситуаций и для ситуаций, предшествующих ликвидации) была полезна, но дополнительная ценность регионального механизма, по сравнению с другими релевантными документами, не была очевидной из-за того, что их содержание частично повторяло друг друга.

**Вывод:**

Региональный механизм положительно повлиял на планирование и осуществление мер по контролю туберкулеза на национальном уровне. В будущем такой региональный механизм должен обрисовывать долговременные стратегические перспективы и конкретно реагировать на возникающие тенденции и старые, хорошо известные проблемы, с которыми сталкиваются страны и районы этого региона.

## Introduction

Tuberculosis remains a major public health concern in the World Health Organization (WHO) Western Pacific Region.^1^ The region has a population of 1.9 billion people with 37 countries and areas, including many Pacific Island countries and areas.^2^ The region accounts for nearly 20% of the global burden of tuberculosis with an estimated 1.8 million incident cases in 2019; 1.4 million (78%) of these cases were reported to national tuberculosis programmes.^1^ In 1999, the WHO Regional Committee for the Western Pacific declared a tuberculosis crisis in the region and the regional office subsequently established the Stop TB Special Project (now called the End TB unit).^3^^,^^4^ The End TB unit has developed three regional strategic plans since the year 2000.^5^^–^^7^

The current *Regional framework for action on implementation of the End TB Strategy in the Western Pacific, 2016–2020* was published in 2016, after extensive consultation with countries and areas, regional experts and international partners engaged in tuberculosis control.^8^ The framework was approved at the Sixty-sixth Regional Committee for the Western Pacific in October 2015 (resolution WPR/RC66.R3),^9^ after the World Health Assembly had approved the new End TB Strategy^10^ (resolution WHA67.1) in May 2014.^11^ The regional framework for action aimed to interpret the concepts of the End TB Strategy in the particular circumstances and contexts of the countries and areas of the Western Pacific Region. Implementation of the framework required: “quality, people-centred TB services for all patients and families, action to address the looming burden of drug-resistant TB, social and financial risk protection to address vulnerability, effective regulatory policies to support TB control efforts, and new tools and capacity for rapid adoption of new technologies”.^8^

The regional framework built on the same three pillars as the End TB Strategy: (i) integrated, patient-centred care and prevention; (ii) bold policies and supportive systems; and (iii) intensified research and innovation. The framework included 16 subtopics linked to proposed actions with defined regional targets and indicators. In view of the diversity of the region, a tiered approach was proposed with actions for all settings, specific settings and pre-elimination settings.^8^ Given that the regional framework defined a strategic vision until 2020, it was timely to review progress in implementation. We therefore aimed to assess implementation of the regional framework, and this paper outlines the main findings of our evaluation.

## Methods

### Study design

We undertook a mixed methods evaluation to assess the implementation of the regional framework and progress towards its targets and indicators. We conducted the evaluation in collaboration with the End TB unit of the WHO Regional Office for the Western Pacific, WHO country offices, national tuberculosis programmes and other stakeholders. The main objectives of the evaluation were to: (i) assess progress against the targets and indicators defined in the regional framework; (ii) determine how the regional framework has been adopted at the country level; (iii) ascertain the perceived value of the regional framework in achieving its objectives; and (iv) describe country-level challenges and success stories, the influence of the regional framework and future challenges. For the first objective, our findings have been reported in a separate paper,^12^ complementing regular epidemiological analyses.^13^ For the second objective, we undertook a review of national tuberculosis strategic plans and other relevant documents, while for the third objective, we conducted a cross-sectional survey of senior tuberculosis programme staff. In addition, we arranged key informant interviews to gather the views of a range of relevant stakeholders on the value of the regional framework, including senior advisers from international donor and technical organizations, tuberculosis programme managers and senior tuberculosis consultants or programme staff in selected countries, which also informed the final objective. Together with data from the epidemiological analyses, information from these interviews formed the basis of country case studies.

We chose this comprehensive mixed methods approach to obtain multiple perspectives and critical real-life insights. We carried out the evaluation (including data collection/interviews) between July and December 2019.

### Data collection and analyses

We developed several evaluation tools which included a data extraction guide for the policy review (objective ii, available in the data repository),^14^ an online cross-sectional survey for senior tuberculosis programme staff (objective iii, available in the data repository),^14^ and an interview guide for in-depth key informant interviews (objectives iii and iv, available in the data repository).^14^ We performed a descriptive analysis of the policy review and calculated numbers and proportions for the cross-sectional survey. We summarized the results from the key informant interviews as a narrative and identified key themes. We developed country case studies using a set template (available in the data repository).^14^

We did not require ethical approval for this quality improvement exercise. Countries and areas voluntarily provide their data to the WHO Global TB Programme, as part of standard reporting practices.

## Results

### National tuberculosis strategic plans

Only 14 of 37 countries and areas of the region had a national tuberculosis strategic plan (Table 1), including all seven priority countries that collectively represent about 95% of the region’s tuberculosis burden, i.e. Cambodia, China, Lao People's Democratic Republic, Mongolia, Papua New Guinea, Philippines and Viet Nam.^9^ Of the other 23 countries and areas, 14 had a national health or development plan that included tuberculosis, while eight had no national health plan that we could find. These eight were all small Pacific Island countries or areas with a low burden of tuberculosis. As well, one country had a tuberculosis guideline, but not a national tuberculosis strategic plan. The 14 countries or areas that had strategic plans accounted for 99.7% of the regional burden of tuberculosis. Table 1 gives a summary of all the strategic plans we reviewed. Of the six strategic plans that had the same 5-year time frame (2016–2020) as the regional framework (Australia, China, Kiribati, Malaysia, Mongolia and Vanuatu), four referred to either the regional framework or the preceding strategy (Kiribati, Malaysia, Mongolia and Vanuatu).^5^ Two strategic plans started in 2017 (Lao People's Democratic Republic and Philippines) with the Lao People's Democratic Republic strategic plan clearly guided by the regional framework. The Australian national tuberculosis strategic plan referenced other WHO documents such as the End TB Strategy^10^ and the framework on elimination of tuberculosis in low-incidence countries.^15^ The Chinese national tuberculosis strategic plan did not reference any WHO documents, although we only reviewed an abbreviated translated version of this plan; however, the Chinese national tuberculosis strategic plan does not usually reference international documents (personal communication, Zhongdan Chen, WHO China Country Office, May 2020). The national tuberculosis strategic plans of Cambodia, Fiji, Japan, Papua New Guinea and Viet Nam all predated the current regional framework, and referred to other WHO documents as well as the millennium development goals (MDGs)^17^ or the sustainable development goals (SDGs).^16^

**Table 1:** Overview of national tuberculosis strategic plans and national health plans identified, WHO Western Pacific Region

Country or area	Type of plan	Name of document	Time period	Reference to regional frameworks		Reference to other relevant WHO documents
				2016–2020^8^	2011–2015^5^	
Australia	Tuberculosis strategic plan	Strategic plan for control of tuberculosis in Australia: towards disease elimination	2016–2020	No	No	End TB Strategy;^10^ Framework towards tuberculosis elimination in low incidence countries^15^
Brunei Darussalam	Health plan	Health system and infrastructure master plan for Brunei Darussalam framework	2016–2035	No	No	SDGs^16^
Cambodia	Tuberculosis strategic plan	National strategic plan for control of tuberculosis	2014–2020	No	Yes	End TB Strategy;^10^MDGs^17^
China	Tuberculosis strategic plan	13th five-year national TB prevention and treatment plan	2016–2020	No	No	Four comprehensive strategic blueprints
Cook Islands	Health plan	Cook Islands national health strategic plan	2017–2021	No	No	Several documents mentioned, but not the End TB Strategy or SDGs
Fiji	Tuberculosis strategic plan	Let’s end TB: Fiji free of TB. Fiji’s response to TB: a national strategy plan	2015–2019	No	No	MDGs^17^
French Polynesia	Health plan	*Schéma de prévention et de promotion de la sante de la Polynésie Française*	2018–2022	No	No	Implied reference to the SDGs^16^ and possibly the End TB Strategy^10^
Japan	Tuberculosis strategic plan	Stop TB action plan	2014–2020	No	No	Global plan to stop TB 2006–2015;^18^End TB Strategy;^10^ SDGs^16^
Kiribati	Tuberculosis strategic plan	National tuberculosis and leprosy strategic plan	2016–2020	Yes	No	End TB Strategy;^10^ SDGs;^16^ Global leprosy strategy: accelerating towards a leprosy-free world 2016–2020^19^
Lao People's Democratic Republic	Tuberculosis strategic plan	National TB strategic plan update 2017–2020	2017–2020	Yes	No	End TB Strategy^10^
Malaysia	Tuberculosis strategic plan	National strategic plan for tuberculosis control	2016–2020	Yes	No	End TB Strategy;^10^ Global tuberculosis report 2016;^20^Toolkit to a develop a national strategic plan for TB prevention, care and control^21^
Marshall Islands	Health plan	Three-year rolling strategic plan	2017–2019	No	No	MDGs;^17^SDGs^16^
Micronesia (Federated States of)	Development plan	Federated States of Micronesia national development plan	2004–2023	No	No	None
Mongolia	Tuberculosis strategic plan	National strategy on strengthening tuberculosis: prevention, care and control	2016–2020	Yes	No	Guidance on ethics of tuberculosis prevention, care and control;^22^Mission reports from the regional Green Light Committee and others (unpublished)
Nauru	Health plan	National health strategic plan	2016–2020	No	No	None
New Caledonia	Health plan	*Plan de Sante Calédonien*	2018–2028	No	No	None
New Zealand	Tuberculosis guideline	Guidelines for tuberculosis control in New Zealand	2019	No	No	End TB Strategy;^10^ SDGs^16^
Niue	Health plan	Health strategic plan	2011–2021	No	No	None
Palau	Health plan	Ministry of health strategic plan	2014–2018	No	No	None
Papua New Guinea	Tuberculosis strategic plan	National tuberculosis strategic plan for Papua New Guinea	2015–2020	No	No	End TB Strategy^10^
Philippines	Tuberculosis strategic plan	Philippine strategic TB elimination plan: phase 1	2017–2022	No	No	End TB Strategy;^10^SDGs^16^
Republic of Korea	Tuberculosis strategic plan	2nd national strategic plan for tuberculosis control	2018–2022	No	No	Global TB Report 2016; End TB Strategy^10^
Samoa	Health plan	Health sector plan	2008–2018	No	No	None
Solomon Islands	Health plan	National health strategic plan	2016–2020	No	No	MDGs;^17^SDGs^16^
Tokelau	Health plan	Tokelau department of health strategic plan	2016–2020	No	No	SDGs^16^
Tonga	Health plan	National health strategic plan	2015–2020	No	No	None
Tuvalu	Health plan	Tuvalu health reform strategy	2016–2019	No	No	MDGs^17^
Vanuatu	Tuberculosis strategic plan	National strategic plan for tuberculosis	2016–2020	No	Yes	End TB Strategy;^10^ SDGs^16^
Viet Nam	Tuberculosis strategic plan	National strategic plan for tuberculosis control for the period 2015–2020	2015–2020	No	No	End TB Strategy;^10^ Regional Green Light Committee reports 2012 and 2013 (unpublished)

MDGs: millennium development goals; SDGs: sustainable development goals; TB: tuberculosis; WHO: World Health Organization.

Note: No relevant strategic plans were identified for American Samoa; Macao Special Administrative Region, China; Guam; Hong Kong Special Administrative Region, China; Commonwealth of the Northern Mariana Islands; Pitcairn Islands; Singapore; and Wallis and Futuna Islands.

The four countries that had national tuberculosis strategic plans that were clearly informed by the regional framework were Kiribati, Lao People's Democratic Republic, Malaysia and Mongolia (Table 2). The specified indicators were generally well aligned with the regional framework, although the catastrophic costs indicator was not included in plans from Kiribati and Mongolia. Most of the proposed actions in the regional framework for all settings were reflected in these strategic plans, but we identified some gaps. For example, actions related to social protection, health in all policies, surveillance and assessment of vital registration systems, and pharmacovigilance were missing from the Mongolian national tuberculosis strategic plan.

**Table 2:** Alignment of national tuberculosis strategic plans with the regional framework^a^

Country	General approach	Alignment with indicators and/or targets	Alignment with proposed actions for^b^	
			All settings	Specific settings
Kiribati	Aligned with the End TB Strategy,^10^ the regional framework^8^ and the global leprosy strategy.^19^ Builds on the previous strategic plan^23^	Mostly: to reduce tuberculosis incidence by 10% and mortality by 35% by 2020, relative to 2015 baselines while maintaining treatment success rate of > 90%	Mostly: some actions are missing including patient cost assessment, surveillance and assessment of the vital registration system, engagement of private sector^c^ and social protection	Mostly: addresses tuberculosis–diabetes co-morbidity, PATLAB and access to quality-assured second-line tuberculosis medicines
Lao People's Democratic Republic	Aligned with the End TB Strategy,^10^ the regional framework^8^ and based on the local epidemiological situation	Fully: 35% reduction in number of tuberculosis deaths compared with 2015; 20% reduction in tuberculosis incidence rate to 146/100 000 population compared with 2015; zero families affected by tuberculosis facing catastrophic costs	Mostly: Some actions are missing including those related to social determinants and poor people, assessment of the surveillance system and management algorithms for latent tuberculosis infection	NA
Malaysia	Adapted from the regional framework,^8^ End TB Strategy,^10^ Global tuberculosis report 2016,^20^ and the toolkit to develop a national strategic plan for tuberculosis prevention, care and control^21^	Partly: 25% reduction in number of tuberculosis deaths compared with 2015; increase in incidence rate of tuberculosis to 100 per 100 000 population compared with 79 per 100 000 in 2015 through enhanced case detection; zero families affected by tuberculosis facing catastrophic costs	Almost fully: very closely aligned with the regional framework including many of the activities adopted from the regional framework	Mostly: addresses tuberculosis in migrants (12–14% of the total tuberculosis caseload). The other proposed actions for specific settings are not relevant to the context of Malaysia
Mongolia	Largely informed by an evaluation of the national tuberculosis strategic plan 2010–2015 although the regional framework was referenced	Mostly: to decrease tuberculosis incidence by 4% and mortality by 30% by 2020 compared with 2014	Mostly: some actions are missing, notably those related to social protection, health in all policies, surveillance and assessment of vital registration system, and pharmacovigilance	NA

NA: not applicable; PATLAB: Pacific TB Laboratory Network.

^a^ *Regional framework for action on implementation of the End TB Strategy in the Western Pacific, 2016–2020*.^8^

^b^ Elimination setting was not relevant to any of the four countries.

^c^ The private sector in Kiribati is small and may have been excluded for this reason.

Note: “Fully” means that the indicators in the national tuberculosis strategic plan were the same three indicators in the regional framework with the same targets. “Mostly” means that some of the indicators were the same (not all three but that there was broad alignment). “Partly” means that fewer indicators were the same (usually only one or two, with less alignment with the regional indicators overall).

Table 3 (available at: http://www.who.int/bulletin/volumes/99/5/20-268060), provides a summary of the main objectives, indicators and targets in the national tuberculosis strategic plans of the seven priority countries of the region. The strategic plans of Cambodia, China, Papua New Guinea, Philippines and Viet Nam were mostly aligned with the WHO End TB Strategy,^10^ as well as other international documents and national or local reports. A summary of the China national strategic plan was translated into English and reviewed for this policy analysis. Therefore, additional detail might have been missed as the full version was not available in English.

**Table 3:** General approach and main indicators and targets in the national tuberculosis plans of the seven high-priority countries in the Western Pacific Region

Country	Period	General approach	Main indicators and targets
Cambodia	2014–2020	Aligned with the End TB Strategy,^10^ the MDGs^17^ and, to a lesser extent, the 2011–2015 regional strategy on tuberculosis in the Western Pacific^5^	Prevalence of bacteriologically positive tuberculosis reduced by 5% a year in people > 15 years; Tuberculosis mortality rate reduced by 5.5% a year in the general population; Tuberculosis incidence rate reduced by 4% a year in the general population
China	2016–2020	Aligned with national strategic blueprints and the End TB Strategy^10^	Nationwide incidence of pulmonary tuberculosis reduced to lower than 58/100 000 population; Tuberculosis incidence in regions with the highest prevalence of tuberculosis reduced by 20% compared with 2015
Lao People's Democratic Republic	2017–2020	Aligned with the End TB Strategy,^10^ and the regional framework^8^	Number of tuberculosis deaths reduced by 35% compared with 2015; Incidence of tuberculosis reduced to 146/100 000 population compared with 2015; Zero families affected by tuberculosis facing catastrophic costs due to tuberculosis
Mongolia	2016–2020	Largely informed by an evaluation of the national tuberculosis strategic plan 2010–2015^24^ although the regional framework^8^ was referenced	Tuberculosis incidence reduced by 4% compared with 2014; Tuberculosis mortality reduced by 30% compared with 2014
Papua New Guinea	2015–2020	Based on the End TB Strategy^10^ and guided by Papua New Guinea vision 2050,^25^ the Papua New Guinea national health plan 2011–2020^26^ and the national tuberculosis strategic plan 2010–2015^27^	Estimated tuberculosis prevalence rate reduced from 541/100 000 population in 2012 to 339 000/100 000 population by 2020; Estimated tuberculosis mortality rate reduced from 54/100 000 population a year in 2012 to 30/100 000 population a year by 2020
Philippines	2017–2022^a^	Primarily based on the End TB Strategy,^10^ and the SDGs^16^	Number of tuberculosis deaths reduced by 50%, from 22 000 to 11 000; Tuberculosis incidence rate reduced by 23%, from 554/100 000 population to 427/100 000; Catastrophic costs to households affected by tuberculosis reduced from 35% to 0%; At least 90% of patients satisfied with the services of the DOTS facilities
Viet Nam	2015–2020	Based on the End TB Strategy,^10^ Regional Green Light Committee report and other national documents	Tuberculosis prevalence rate in the community reduced to 131/100 000 population, from 218/100 000 in 2012; Tuberculosis mortality rate reduced to less than 10 deaths/100 000 population, from 20/100 000 in 2012; Incidence rate of multidrug-resistant tuberculosis kept at less than 5% of total new tuberculosis cases

DOTS: directly observed treatment, short-course; MDGs: millennium development goals; SDGs: sustainable development goals; TB: tuberculosis.

^a^ In the Philippines, the period of the national tuberculosis strategic plan (i.e. 2017–2022) is aligned with the term of the current government

### Survey of senior staff

We received 23 of 37 completed surveys from senior staff members of national tuberculosis programmes and 19 respondents indicated that they had read the regional framework or part of it. Of these 19 respondents, 16 found the regional framework helpful in the development of their national tuberculosis strategic plan. Eleven of 22 respondents said that their national tuberculosis strategic plans were wholly or greatly guided by previous or current regional strategies. Other documents identified by respondents that guided the development of national tuberculosis strategic plans included: national development plans; national surveys and reports; the End TB Strategy; the MDGs; the SDGs; documents from the United States Centers for Disease Control and Prevention; the results of operational research; and other WHO documents including *Implementing the End TB Strategy: the essentials*^28^ and *Towards tuberculosis elimination: an action framework for low-incidence countrie*s.^15^

Respondents indicated that the distinction between guidance for all, specific and pre-elimination settings was useful and ensured that the regional framework could serve as a resource for all countries and areas. Programmatic hurdles that the respondents thought needed to be considered when planning technical assistance or developing future regional frameworks included financing, human resources capacity, public–private partnership, active case finding, management of paediatric and drug-resistant tuberculosis and general health system challenges. Of 12 programmatic areas assessed, the senior tuberculosis staff thought that the regional framework provided the most relevant guidance on the treatment and care of adult tuberculosis patients, the development of adequate laboratory capacity to guide treatment of drug-resistant cases, and treatment and care of children with tuberculosis (Table 4).

**Table 4:** Influence of the regional framework^a^ on various areas of tuberculosis control in countries and areas of the WHO Western Pacific Region

Area of tuberculosis control	Responses, no. (%)					Total no. of responses	Weighted average^c^
	Scale^b^						
	1	2	3	4	5		
Treatment and care for drug-resistant and drug-susceptible patients	1 (5)	1 (5)	3 (14)	11 (50)	6 (27)	22	3.91
Treatment and care for tuberculosis in children	0 (0)	1 (4)	8 (35)	10 (43)	4 (17)	23	3.74
Treatment and care for tuberculosis and co-morbidities	1 (5)	1 (5)	6 (27)	11 (50)	3 (14)	22	3.64
Treatment and care for tuberculosis in high-risk populations	3 (13)	1 (4)	4 (17)	11 (48)	4 (17)	23	3.52
Strong laboratory networks to find all causes	1 (5)	1 (5)	5 (23)	11 (50)	4 (18)	22	3.73
Strong laboratory capability to guide treatment of drug-resistant cases	1 (4)	1 (4)	5 (22)	11 (48)	5 (22)	23	3.78
Diagnosis and treatment of latent tuberculosis infection	2 (9)	1 (5)	5 (23)	12 (55)	2 (9)	22	3.50
Governance and stewardship (including strategic plans, financing, drug regulation and management, and surveillance)	1 (5)	1 (5)	5 (23)	13 (59)	2 (9)	22	3.64
Engagement and partnerships (including patients, civil society and all care providers, including the private sector)	3 (14)	2 (9)	5 (23)	10 (45)	2 (9)	22	3.27
Addressing social protection, poverty and social determinants of health	3 (14)	2 (10)	6 (29)	9 (43)	1 (5)	21	3.14
Enhancing tuberculosis research capacity for development, rapid update and optimum use of new interventions	2 (9)	4 (18)	4 (18)	10 (45)	2 (9)	22	3.27
Other areas	2 (15)	0(0)	6 (46)	5 (38)	0 (0)	13	3.08

^a^ *Regional framework for action on implementation of the End TB Strategy in the Western Pacific, 2016–2020*.^8^

^b^ 1 = no positive influence to 5 = very strong positive influence.

^c^ A self-weighted average was calculated by the survey software (Survey Monkey®, San Mateo, United States of America).

Note: Respondents were senior staff of national tuberculosis programmes.

### Key informant interviews

We contacted 24 key informants for critical reflection on the value of the regional framework and we interviewed 17 of the informants contacted. Interviewees were with the main organizations and technical agencies working on tuberculosis control in the region and high-level national tuberculosis programme representatives in certain countries (list of organizations and agencies available in the data repository).^14^ Respondents thought that the regional framework had value, but considered that it duplicated the content of other WHO documents. Therefore, its added value compared with existing WHO documents required careful consideration. Interviewees had mixed responses on whether national tuberculosis programme targets were influenced by the regional framework. Specific examples where the targets differed included Viet Nam, which defined a prevalence (instead of incidence) target; Australia. which did not define a mortality target; and many countries which failed to include targets for catastrophic cost. Grants from the Global Fund to Fight AIDS, Tuberculosis and Malaria are usually aligned with the targets in national tuberculosis strategic plans and therefore respondents felt that the regional framework had an indirect influence on Global Fund grant programming through its influence on national tuberculosis strategic plans. Most interviewees thought that providing particular actions for all settings, specific settings and pre-elimination settings was useful, but some actions were seen to be more achievable than others. Selected quotes about the perceived value of the regional framework and the relevance of proposed actions are included in the data repository[Boxed-text B1].^14^ To inform the development of a future regional framework or a similar strategic planning document, interviewees were asked about successes and challenges since the regional framework was released (Box 1). Generally, interviewees thought that a revised regional framework, and the necessary accompanying effort to launch and promote a framework, would help to keep tuberculosis on political agendas across the region.

Box 1 **Successes and challenges of national tuberculosis programmes mentioned by key informants, WHO Western Pacific Region**

***Detect***

*Successes*: strong focus on active case finding; general laboratory improvement and expanded networks for drug-resistant tuberculosis diagnosis and treatment, including the use of Xpert Ultra®, line probe assay and whole genome sequencing; low number of multidrug-resistant tuberculosis cases among new cases

*Remaining challenges*: maintenance of Xpert^®^ MTB/RIF equipment and subsequent loss of skills in smear microscopy; scaling up of detection and treatment of paediatric tuberculosis; low case finding and treatment success for drug-resistant tuberculosis; inadequacy of efforts to find missing cases

***Treat***

*Successes*: removal of the category two regimen; availability of child-friendly water-dispersible fixed-dose combination tablets; better patient support to reduce catastrophic costs; implementation of short-course regimens for management of MDR and rifampicin-resistant tuberculosis; access to bedaquiline and delamanid as required

*Remaining challenges*: poor patient-centred care (still a paternalistic approach); scaling up of detection and treatment of drug-resistant tuberculosis with high loss to follow-up; high cost of some medicines for multidrug-resistant tuberculosis which are not included in national drug lists and low treatment success

***Prevent***

*Successes*: better awareness of tuberculosis prevention and new WHO guidelines on infection prevention and control

*Remaining challenges*: screening, prevention and treatment of latent tuberculosis infection; lack of commitment to provide preventive therapy to household contacts in high-incidence settings, even to vulnerable children

***Recording and reporting***

*Successes*: on track to reach targets but much left to do; relatively low caseload of multidrug-resistant tuberculosis

*Remaining challenges*: improvement in documentation to obtain funding; strengthening of surveillance and use of data for field-based research to provide scientific evidence for policy-making

***Research and innovation***

*Successes*: more and better quality research undertaken, e.g. inventory studies, patient cost surveys, new diagnostics

*Remaining challenges*: strengthening of research capacity and securing funding

***Partnerships and collaboration***

*Successes*: improved collaboration and better partnerships, and community engagement; improved private sector and donor engagement; more collaboration with other groups on tuberculosis care (e.g. civil society, nongovernmental organizations)

*Remaining challenges*: still insufficient engagement of the private and non-public sector; insufficient engagement of civil society in tackling tuberculosis; overwhelming of national programmes by international partners

***Health systems***

*Successes*: increased funding from government; health system improvements, e.g. decentralized care; more attention on universal health coverage

*Remaining challenges*: difficulty in using funding; regulatory barriers, e.g. excessive bureaucracy; strengthening of health-care delivery system; better trained human resources; large funding gaps for national tuberculosis plans

***WHO regional office***

*Successes*: improved commitment and top-level engagement after the UN high-level meeting on tuberculosis in 2018

*Remaining challenges*: better cross-regional collaboration and harmonization between the WHO Western Pacific and South-East Asia regions; maintaining political advocacy at the top level following the UN high-level meeting

MDR: multidrug-resistant; UN: United Nations; WHO: World Health Organization.Notes: Each success or challenge does not necessarily apply to all the countries and some refer to the region more broadly. Key informants were senior advisers from international donor and technical organizations, tuberculosis programme managers and senior tuberculosis consultants or programme staff in selected countries.

### Country case studies

**Fig. 1:**
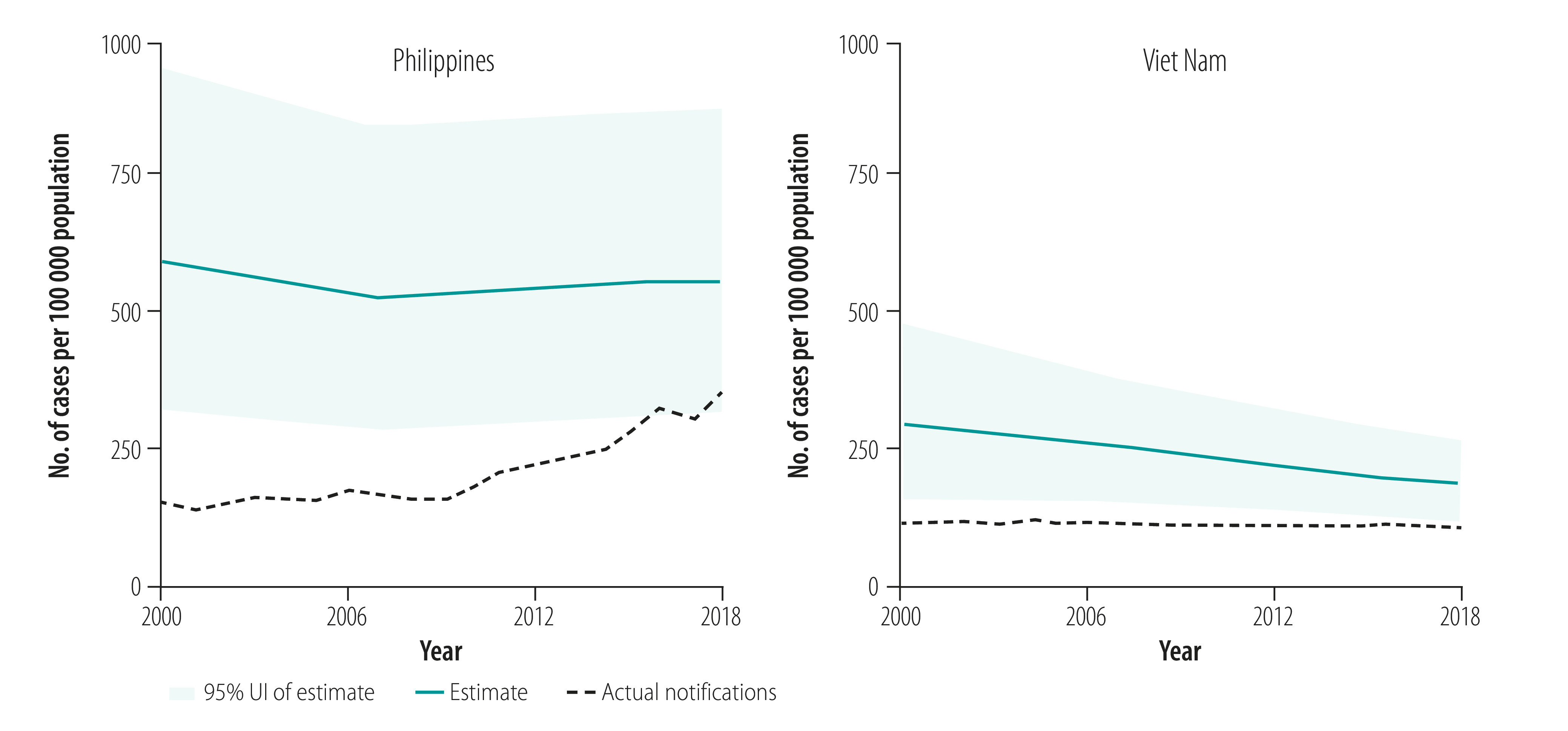
Incidence of tuberculosis, Philippines and Viet Nam, 2000–2018

**Fig. 2:**
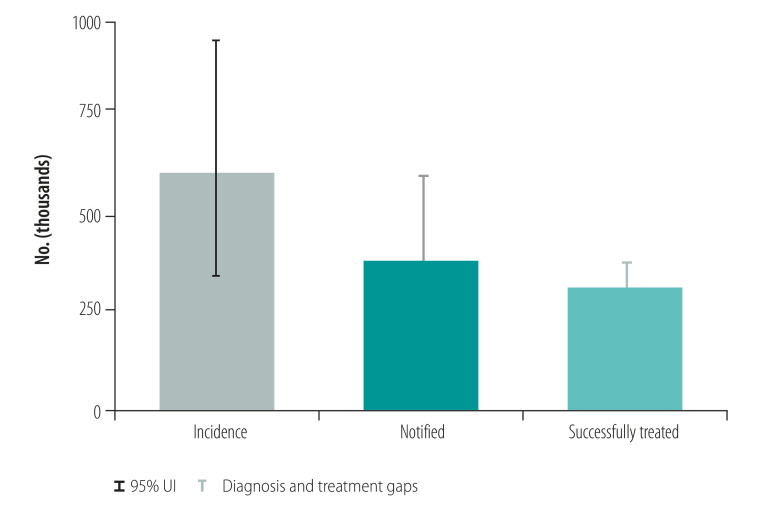
Treatment of tuberculosis, Philippines, 2018

**Fig. 3:**
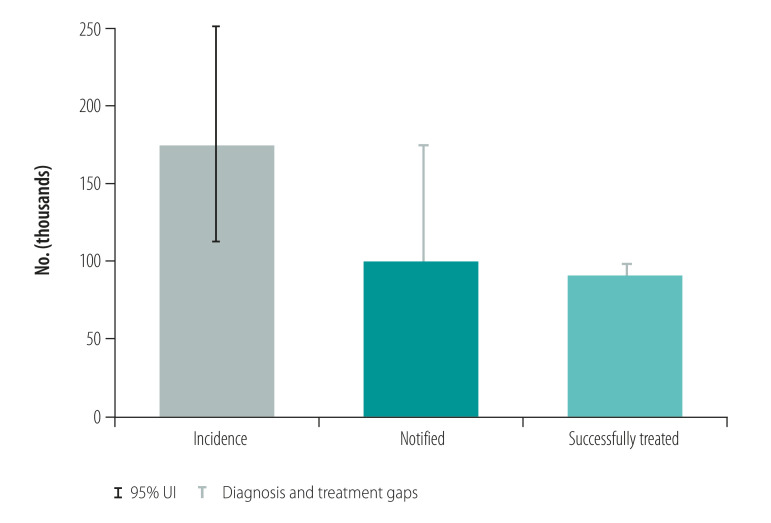
Treatment of tuberculosis, Viet Nam, 2018

**Fig. 4:**
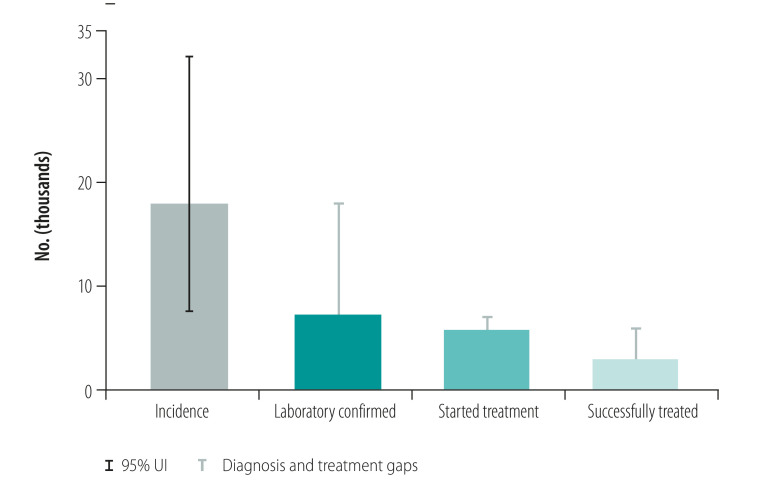
Treatment of multidrug- and rifampicin-resistant tuberculosis, Philippines, 2018

**Fig. 5:**
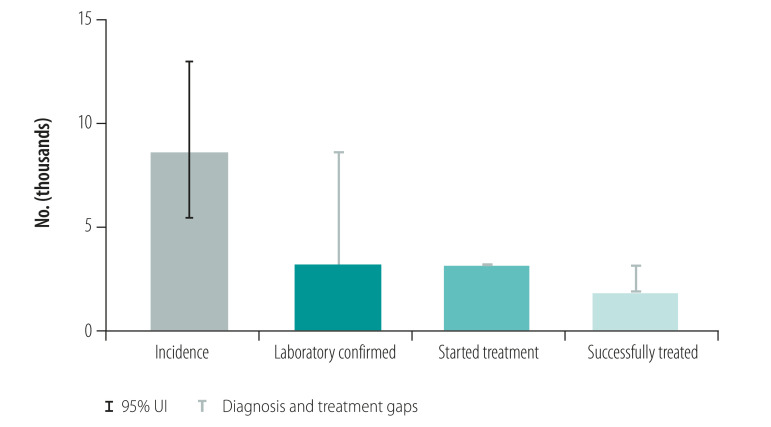
Treatment of multidrug- and rifampicin-resistant tuberculosis, Viet Nam, 2018

Countries included in the case studies were Australia, Cambodia, China, Papua New Guinea, Philippines, Solomon Islands and Viet Nam. The focus of the cases studies was on key lessons learnt. Tuberculosis control in Papua New Guinea and the Philippines was facing significant challenges such as rising numbers of cases of multidrug-resistant tuberculosis and cases in children, while achievements such as productive research collaborations and a new social protection scheme were noted in Viet Nam. Fig. 1 shows the estimated incidence of tuberculosis and actual notified tuberculosis cases in the Philippines and Viet Nam from 2000 to 2018. The Philippines has one of the highest rates of tuberculosis (estimated tuberculosis incidence 554/100 000 population in 2018) with large numbers of patients (382 543 case notifications in 2018)^29^ and evidence of tuberculosis transmission, usually demonstrated by high rates of tuberculosis in children and, when available, whole genome sequencing.^30^ The Philippines has intensified active case finding activities among high-risk groups and vulnerable populations and has scaled up molecular diagnostic tests in line with the End TB Strategy and the regional framework. Therefore, the case notification rate has risen in recent years (Fig. 1). However, these gains may be undermined by issues such as inadequate funding, potential transmission of tuberculosis in prisons and other settings where large numbers of people are gathered, and the recent adverse effect of coronavirus disease 2019 (COVID-19). Viet Nam has shown substantial progress towards the targets of the End TB Strategy with high-level political commitment to tuberculosis control efforts, productive research collaborations and intensified social protection schemes. Aligned with the End TB Strategy and the regional framework, molecular diagnostic tests and new regimens for drug-resistant tuberculosis have also been rapidly scaled up. However, in both the Philippines and Viet Nam, considerable gaps remain between estimated incidence and diagnosis of tuberculosis, with smaller gaps between diagnosis and treatment (Fig. 2 and Fig. 3). For multidrug- and rifampicin-resistant tuberculosis, the gaps between estimated incidence and diagnosis are also considerable and the Philippines reports a gap between cases diagnosed and those treated (Fig. 4 and Fig. 5). 

UI: uncertainty interval.

UI: uncertainty interval.

UI: uncertainty interval.

UI: uncertainty interval.

Note: Data for 2017 were used for successfully treated cases

UI: uncertainty interval.

Note: Data for 2017 were used for successfully treated cases

## Discussion

The framework aimed to assist the translation of the End TB Strategy to the national and regional context in the WHO Western Pacific Region. As such, the framework outlined ambitious tuberculosis targets for the region aligned with the End TB Strategy^10^ and the SDGs:^8^ a 95% reduction in tuberculosis deaths, a 90% reduction in tuberculosis incidence and zero catastrophic costs for households affected by tuberculosis by 2035 compared with 2015. However, the Western Pacific Region is not on track to meet these targets, given that the estimated tuberculosis incidence and mortality rates have decreased by only 3% and 10% respectively since 2015, whereas the regional framework targets for 2020 were a 20% reduction in tuberculosis incidence and 35% reduction in mortality.^8^^,^^10^ In the Western Pacific Region, tuberculosis treatment coverage (case detection) for drug-susceptible tuberculosis is currently 78% (1.4/1.8 million), indicating that about 400 000 cases are untreated every year; the largest case detection gap is in children under 5 years.^29^ In 2018, 72% (72 216/101 000) of the estimated incident cases of drug-resistant tuberculosis were missed.^29^ The rise in drug-resistant tuberculosis, coupled with an ageing population,^31^ widespread undernutrition^32^ and increased co-morbidities such as diabetes^33^ and smoking-related lung disease,^34^ are important challenges to regional tuberculosis control efforts. Thus, achieving the milestones and targets of the regional framework will require urgent implementation of specific actions in line with the principles of the End TB Strategy and the regional framework.^10^^,^^28^^,^^35^

The success of any global or regional tuberculosis strategy is based on its translation into national strategic plans and actual on-the-ground implementation.^21^ National tuberculosis strategic plans were key documents in this evaluation as they define the vision, goal and objectives of tuberculosis control efforts in a country and are a key tool for resource mobilization. Our evaluation showed that all seven priority countries, five of which also have a high tuberculosis burden, have a national tuberculosis strategic plan. In general, the regional framework provided valuable guidance to countries and areas drafting their strategic plans, but the timing of its release and short time frame (many completed their national tuberculosis strategic plan before its release), inadequate marketing and perceived duplication of content with existing WHO documents may have limited its influence and positive impact.

Our analyses showed substantial gaps in case finding and access to treatment in all country case studies, especially for people with drug-resistant tuberculosis. The need for focused strategies to tackle tuberculosis in high-risk subpopulations was highlighted during key informant interviews. In 2018, WHO, the Stop TB Partnership and the Global Fund launched a joint initiative (FIND. TREAT. ALL. #ENDTB) to urgently scale up tuberculosis case finding.^36^ This initiative aims to diagnose, treat and report 40 million people with tuberculosis between 2018 and 2022, including 3.5 million children and 1.5 million people with drug-resistant tuberculosis, in line with the targets set at the UN high-level meeting on tuberculosis.^36^^,^^37^ Countries and areas of the Western Pacific Region are encouraged to join these efforts and take action to close these case detection gaps and promote access to care.

Responses from national tuberculosis programme managers and key informants highlighted several areas to consider in a future regional framework. Several technical and programmatic problems were highlighted including laboratory capacity, large-scale case finding for active tuberculosis, drug-resistant tuberculosis care, scaling up of preventive therapy and a lack of national research capacity to address key research questions specific to the country context. Health system challenges noted included the need for universal health coverage (UHC) and the integration of tuberculosis services into primary health care, especially for children, with adequate financing mechanisms in place.^35^^,^^38^ A future regional framework will need to be aware of these programmatic areas while accommodating the varied needs of countries and areas within the region. A regional framework should also include recommendations on accountability at the national, regional and global levels, given that WHO has now developed a multisectoral accountability framework,^39^ which was an outcome of the United Nations high-level meeting on tuberculosis in 2018.^40^ However, since our evaluation was performed, the global COVID-19 pandemic^41^ has had substantial implications on national disease control programmes, including national tuberculosis programmes,^42^^,^^43^ and regaining the lost momentum will take time and effort.

Of the seven priority countries of the Western Pacific Region, Papua New Guinea and the Philippines have the biggest challenge because of large numbers of undetected cases and health system inefficiencies.^29^ Strong political leadership and highly functioning partnerships will be required to improve the tuberculosis situation in these countries. Some small Pacific Island countries (such as Kiribati, the Marshall Islands and Tuvalu) are hotspots for tuberculosis (high rates of tuberculosis and evidence of transmission), although absolute case numbers are small.^29^ Given their geographical isolation and the absence of drug-resistant disease, these countries could potentially serve as positive models for tuberculosis elimination. Population-based approaches for active case finding developed in the Marshall Islands^44^ and Viet Nam^45^ could serve as a template for island-wide elimination strategies, potentially screening for both active tuberculosis disease and latent tuberculosis infection, along with other diseases that may benefit from a similar approach.

Our evaluation has some strengths and limitations. We used both quantitative and qualitative approaches and explored the perceptions of national tuberculosis programme managers and key informants. However, the scope of our evaluation was broad and captured mainly people’s opinions: validation of findings was limited, except in some country case studies. Furthermore, we were unable to validate the specific effect of the regional framework on policies and activities. We also found some inconsistencies between the different components of the evaluation; for example, in the survey of national tuberculosis programme managers, most respondents said that their national tuberculosis strategic plan was influenced by the regional framework, but only four countries published new national tuberculosis strategic plans after the regional framework. Managers might have thought that the regional framework would have influenced their national tuberculosis strategic plans if it had been available at the time, or that it is influencing current plans in development. Determining the real effects of the regional frameworks and strategies is challenging given the large number of priorities, needs, strategies, documents, organizations and funders that influence disease control programming. Although the scope of our evaluation did not cover all aspects of policy development and implementation, preparation of the regional framework included comprehensive consultation with countries and areas, and the formation of a regional tuberculosis technical advisory group has provided a strong platform to develop, disseminate and monitor future regional frameworks.

Overall, the regional framework has had a positive influence on national tuberculosis programmes in the Western Pacific Region, where tuberculosis incidence and mortality are slowly declining. The findings of our evaluation will inform the development of a future regional framework. This framework will be aligned with the priorities of countries and areas, the *Thirteenth general programme of work, 2019–2023* of WHO, and the document: *For the future: delivering better health in the Western Pacific Region*.^46^^,^^47^ WHO’s thirteenth General Programme of Work is structured around three interconnected priorities linked to tuberculosis care: achieving UHC; addressing health emergencies; and promoting healthier populations, with targets of reaching 1 billion people in each thematic area.^46^ In line with the triple billion theme, the vision for the WHO Western Pacific Region is summarized in the report *For the future: towards the healthiest and safest region*.^48^ The priority areas for the region are: health security and antimicrobial resistance; noncommunicable diseases and ageing; environment and living conditions; and reaching the unreached.^48^ Tuberculosis is linked to all four of these priorities and the next regional framework should reflect these links, with careful consideration of how to minimize duplication and maximize added value.
